# Dual Role of Thrombospondin-1 in Flow-Induced Remodeling

**DOI:** 10.3390/ijms222112086

**Published:** 2021-11-08

**Authors:** Céline Grenier, Antoine Caillon, Mathilde Munier, Linda Grimaud, Tristan Champin, Bertrand Toutain, Céline Fassot, Olivier Blanc-Brude, Laurent Loufrani

**Affiliations:** 1 UMR CNRS 6015, 49100 Angers, France; celine.grenier86@gmail.com (C.G.); antoine.caillon.etud@gmail.com (A.C.); mathilde.munier@univ-angers.fr (M.M.); linda.grimaud@univ-angers.fr (L.G.); tristan.champin@gmail.com (T.C.); bertrand.toutain@univ-angers.fr (B.T.); celine.fassot@inserm.fr (C.F.); 2 INSERM U1083, 49100 Angers, France; 3 MITOVASC Institute, University of Angers, 49100 Angers, France; 4 UMR Inserm 970 ParCC-Paris Cardiovascular Center HEGP, 75015 Paris, France; Olivier.Blanc-Brude@inserm.fr

**Keywords:** thrombospondin-1, blood flow, remodeling, resistance arteries, immune cells

## Abstract

(1) Background: Chronic increases in blood flow, as in cardiovascular diseases, induce outward arterial remodeling. Thrombospondin-1 (TSP-1) is known to interact with matrix proteins and immune cell-surface receptors, but its contribution to flow-mediated remodeling in the microcirculation remains unknown. (2) Methods: Mesenteric arteries were ligated in vivo to generate high- (HF) and normal-flow (NF) arteries in wild-type (WT) and TSP-1-deleted mice (TSP-1^−/−^). After 7 days, arteries were isolated and studied ex vivo. (3) Results: Chronic increases in blood flow induced outward remodeling in WT mice (increasing diameter from 221 ± 10 to 280 ± 10 µm with 75 mmHg intraluminal pressure) without significant effect in TSP-1^−/−^ (296 ± 18 to 303 ± 14 µm), neutropenic or adoptive bone marrow transfer mice. Four days after ligature, pro inflammatory gene expression levels (CD68, Cox2, Gp91phox, p47phox and p22phox) increased in WT HF arteries but not in TSP-1^−/−^ mice. Perivascular neutrophil accumulation at day 4 was significantly lower in TSP-1^−/−^ than in WT mice. (4) Conclusions: TSP-1 origin is important; indeed, circulating TSP-1 participates in vasodilation, whereas both circulating and tissue TSP-1 are involved in arterial wall thickness and diameter expansion.

## 1. Introduction

Chronic changes in blood flow occur in physiological conditions such as exercise [1], pregnancy [2] or post-natal development and in pathological conditions such as arterial occlusive diseases [3], diabetes [4] or hypertension [5]. Chronic increase in blood flow induces a remodeling of the arterial wall in order to adapt the wall strain to the new hemodynamic conditions, as described in large blood vessels [6] and small resistance arteries [7]. 

In large vessels (i.e., carotid arteries), chronic increase in blood flow induces limited vasodilatation [6] and parietal hypertrophy [8]. In resistance arteries, a chronic rise in blood flow triggers an increase in diameter associated with medial thickening [7]. Although the mechanism involved in vascular diameter adaptation is well described in large arteries, pathways implicated in arterial remodeling of resistance arteries are less described. Major differences may, however, be expected due to dissimilar vascular wall constitution and specific local hemodynamic parameters.

We have previously shown that pharmacological NO-synthesis blockade or genetic deletion of eNOS in mice prevented high flow (HF)-mediated diameter enlargement in resistance arteries, without effect on hypertrophy [9]. In addition, we have demonstrated that the compensatory wall thickening occurring after a chronic rise in shear stress in HF arteries depends on angiotensin II type 1 receptor stimulation [10] but also on AT2R dependent interleukin-17 production by T lymphocytes [11].

Other studies have demonstrated a relationship between blood flow and thrombospondin-1 (TSP-1) gene expression/protein production [12]. TSP-1 is a matricellular protein with multiple properties, including the modulation of NO signals [13], immune cell recruitment and activation. TSP-1 was first discovered in platelets [14,15], endothelial cells [16], vascular smooth muscle cells [17] and in lymphoid or myeloid cells [18,19]. Its receptor, CD47, is expressed by T [20] and B cells [21], monocytes [22], platelets [23], erythrocyte precursors [24], vascular endothelial cells [25] and vascular smooth muscle cells [26]. Moreover, it has been demonstrated that TSP-1 endothelial expression is stimulated by hypoxia [27] and low NO [28]. More specifically, TSP-1 expression, autocrine and paracrine TSP-1 signaling in vascular cells as CD47 expression are stimulated by sudden changes in shear stress [12,29]. 

It has been previously established that vascular inflammation in resistance arteries subjected to high flow was responsible for diameter enlargement and perivascular macrophage accumulation (2 days) preceding vascular wall remodeling. Macrophage accumulation is observed in mesenteric arteries upon chronic blood flow increase [30,31] and outward hypertrophic remodeling does not occur after macrophage depletion [31]. The contribution of immune cells to many cardiovascular diseases is now recognized, such as in ischemic diseases [32], hypertension [33], uterine under perfusion with preeclampsia [34] or intra-uterine growth restriction [35] and stroke [36,37]. However, their role in outward hypertrophic remodeling remains to be clarified.

The aim of this study is to identify the early triggers of vascular remodeling in resistance arteries and the first stimuli at the origin of inflammatory recruitment and oxidative stress in the acute phase of blood flow increasing [38].

Although the implication of immune cells in flow-induced remodeling has been partly described, the early mechanism of immune cells recruitment and the ignition of the inflammation remains unknown. A better understanding of remodeling mechanisms may provide new therapeutic opportunities for pathologies associated to flow disturbance and many ischemic disease settings [39]. 

## 2. Results

### 2.1. TSP-1 Modulates Flow-Mediated Remodeling

**Figure 1:**
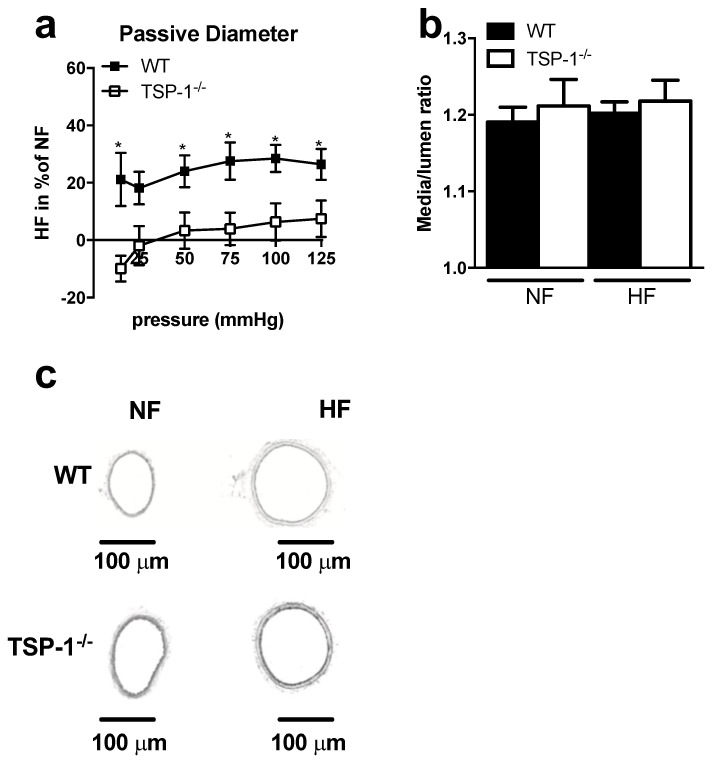
Remodeling of TSP-1^−/−^ and WT mesenteric arteries after seven days of chronic increase in blood flow. Graphs show (**a**) the passive diameter of HF arteries in percent of NF arteries and (**b**) the histomorphometry analysis as media-to-lumen ratio. TSP-1^−/−^ (*n* = 7) mesenteric arteries are represented in white and WT (*n* = 5) in black. (**c**) shows representative images (×10 magnification, scale bar = 100 µm). All results are expressed in means ± sem. * *p* < 0.05, WT vs. TSP-1^−/−^.

In WT mice, the diameters of arteries submitted to a chronic increase in flow (high flow, HF) were greater than the diameters of control arteries under normal flow, NF (Figure 1a,c). This increase in diameter in HF arteries was accompanied by significant wall thickening and cross-section area increase in WT mice (Figure S3a,b), evidenced by the absence of significant changes in media-to-lumen ratios (Figure 1b,c). In TSP-1^−/−^ mice, the diameters of HF arteries failed to expand (Figure 1a,c); their media-to-lumen ratios remained constant (Figure 1b,c) as the cross-section area (Figure S3b).

### 2.2. Circulating TSP-1 Induces Arterial Diameter Enlargement, but Tissue TSP-1 Participates in Arterial Parietal Thickening 

**Figure 2:**
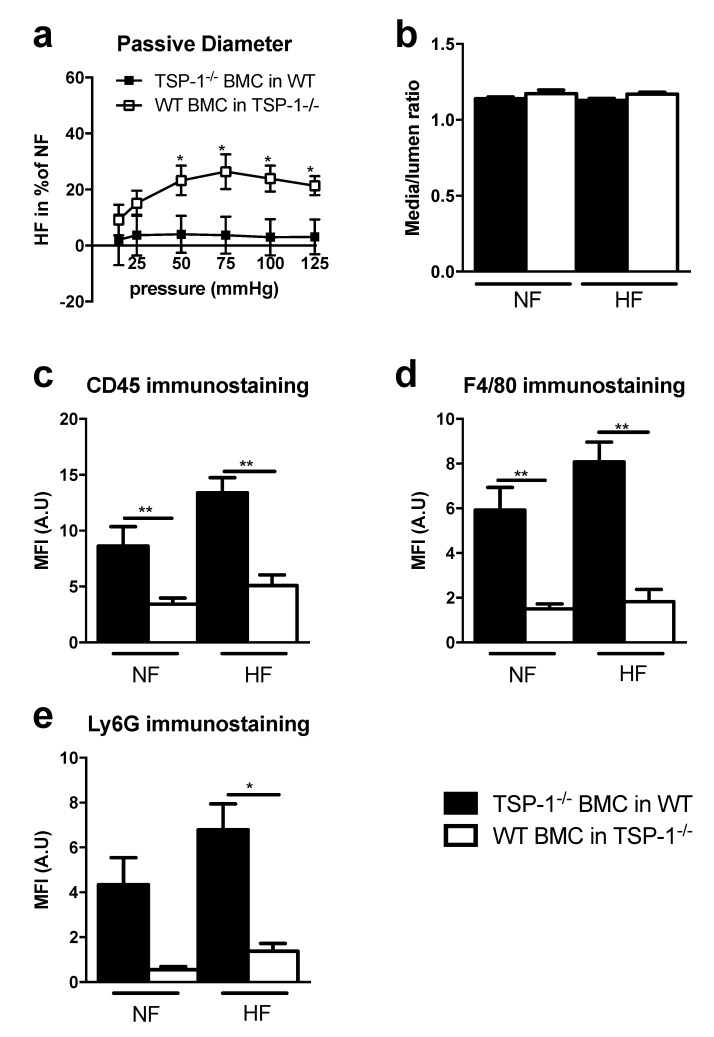
Remodeling study after seven days of chronic increase in blood flow and BMC transfer experiments. Graphs show (**a**) the passive diameter of HF mesenteric arteries in percent of NF mesenteric arteries diameter, (**b**) histomorphometry analysis of these arteries as media-to-lumen ratio. TSP-1^−/−^ BMC in WT, *n* = 9 and WT BMC in TSP-1^−/−^, *n* = 9. The mean fluorescence intensity (MFI) of the peri arterial zone representing the total immune cells (**c**), the macrophages (**d**) and the neutrophils (**e**) is measured after four days of ligature in TSP-1^−/−^ BMC in WT (*n* = 5) and in WT BMC in TSP-1^−/−^ (*n* = 7). TSP-1^−/−^ BMC in WT arteries are represented in black and the WT BMC in TSP-1^−/−^ in white. All results are expressed in means ± sem. * *p* < 0.05, ** *p* < 0.01, TSP-1^−/−^ BMC in WT vs. WT BMC in TSP-1^−/−^.

To determine if the origin of the TSP-1 implicated in HF remodeling was from tissue or circulating cells, we have made bone marrow cells (BMC) transfer after full body irradiation, to generate circulating cells from WT or TSP-1^−/−^ mice in the other genotype. Indeed, we grafted to define two groups:

(1) We transferred WT bone marrow cells to TSP-1^−/−^ mice to have WT circulating cells in TSP1^−/−^ mice. The TSP-1 is only present in circulating cells but not in mice tissues. This group is named “WT BMC in TSP-1^−/−^“.
(2) We transferred TSP-1 bone marrow cells to WT mice to have TSP-1^−/−^ circulating cells in WT mice. So, TSP-1 is only present in mice tissues but not in circulating cells. This group is named “TSP-1^−/−^ BMC in WT“.
  Seven days after ligation, the HF/NF diameter ratio of mesenteric arteries in WT BMC in TSP-1^−/−^ was increased compared to TSP-1^−/−^ BMC in WT.
  Strikingly, there was no significant difference in TSP-1^−/−^ BMC in the WT mice group (Figure 2a), showing a curve belonging to 0% of change. The two curves that were statistically different demonstrated that circulating WT cells, i.e., expressing TSP-1, were necessary for HF diameter expansion.
  Histomorphometry analysis revealed that media/lumen ratio (Figure 2b) and the cross-section area (Figure S3d) were not significantly different in NF and HF arteries of TSP-1^−/−^ BMC in WT and WT BMC in TSP-1^−/−^. Media thickness was significantly increased in HF arteries in WT BMC in TSP-1^−/−^ compared to TSP-1^−/−^ BMC in WT. Furthermore, no differences were found between NF and HF arteries in TSP-1^−/−^ BMC in WT or in WT BMC in TSP-1^−/−^ (Figure S3c).

The study of the immune cell infiltration by confocal microscopy showed that the mean fluorescence intensities of immunostaining for CD45 (Figure 2c), F4/80 (Figure 2d) and Ly6G (Figure 2e) were decreased in both NF and HF arteries of WT BMC in TSP-1^−/−^ compared to TSP-1^−/−^ BMC in WT, meaning that tissue-secreted TSP-1 is important for proper immune cell infiltration.

### 2.3. Hydralazine Does Not Improve Flow-Induced Remodeling in TSP-1^−/−^ Mice

**Figure 3:**
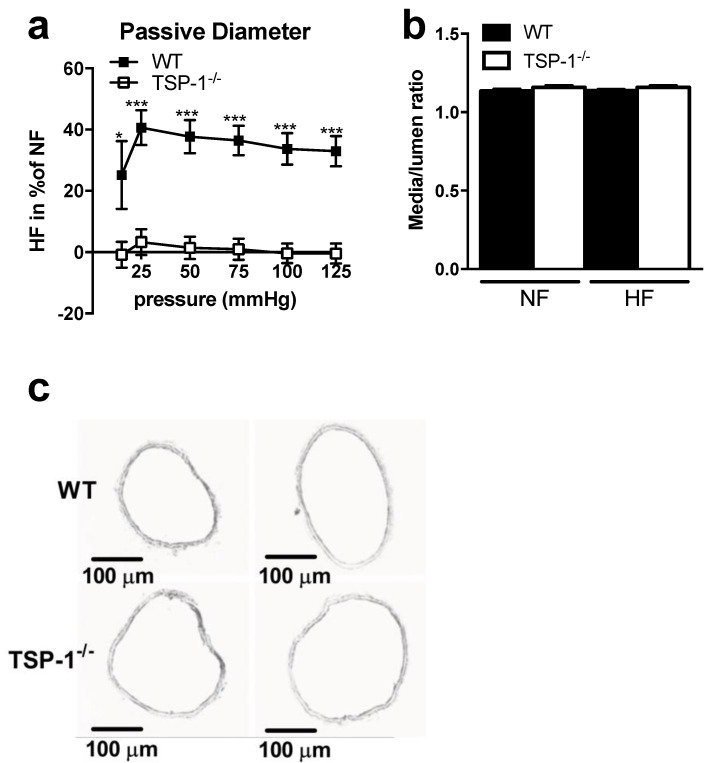
Remodeling study after seven days of chronic increase in blood flow and hydralazine treatment. Graphs show (**a**) the passive diameter of the HF mesenteric arteries in percentage of the NF mesenteric arteries diameter, (**b**) histomorphometry analysis of these mesenteric arteries as media-to-lumen ratio. (**c**) shows representative images (×10 magnification, scale bar = 100 µm). TSP-1^−/−^ (*n* = 10) mesenteric arteries are represented in white and WT (*n* = 6) in black. All results are expressed in means ± sem. * *p* < 0.05, *** *p* < 0.001, WT vs. TSP-1^−/−^.

The non-selective vasodilator hydralazine did not significantly affect HF-remodeling in WT mice and failed to improve it in TSP-1^−/−^ animals (Figure 3). Furthermore, the non-selective vasodilator hydralazine did not improve media thickness or cross section area in TSP1^−/−^ mice (NF vs. HF, Figure S3 e,f), suggesting that a change in shear stress sensitivity is not responsible for the absence of remodeling in TSP-1^−/−^ mice.

### 2.4. TSP-1 Mediates Flow-Induced Immune Cell Recruitment 

**Figure 4:**
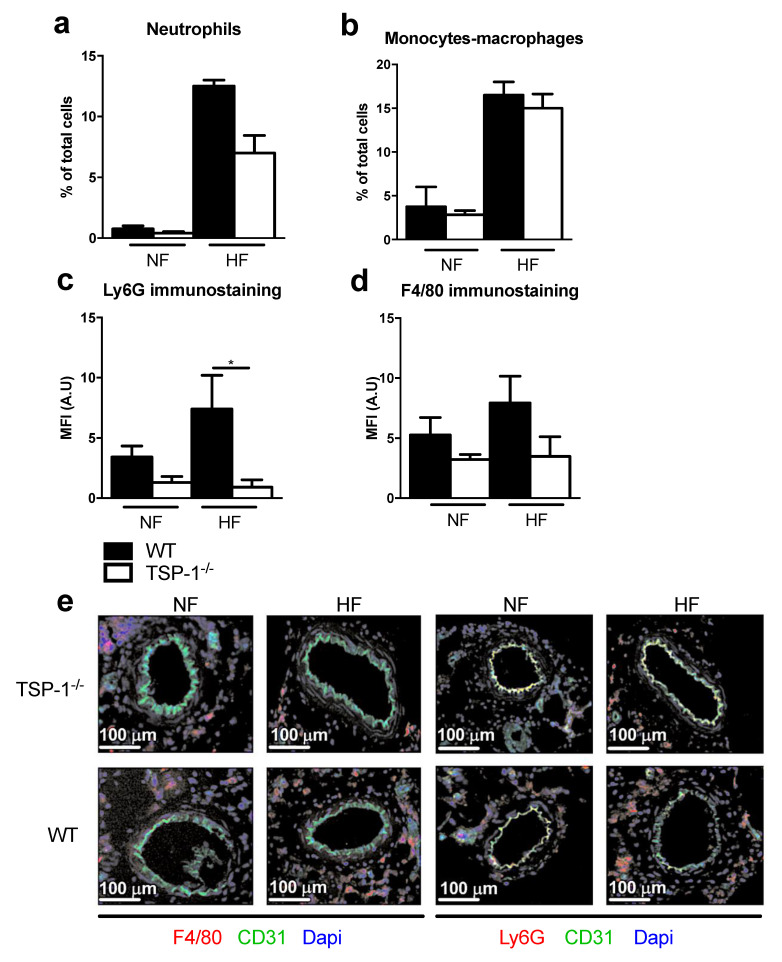
Immune cells recruitment in WT and TSP-1^−/−^ mesenteric arteries after four days of chronic increase in blood flow. Immune cells recruitment study with flow cytometry experiment (WT *n* = 2, TSP-1^−/−^ *n* = 6) results showing neutrophils (**a**) and monocyte and macrophages (**b**) proportion. Mean fluorescence intensity (MFI) quantification of neutrophils (**c**) and macrophages (**d**). (**e**) Representative pictures of mesenteric arteries section immunostained with anti F4/80-PE, anti Ly6G-PE, anti CD31-FITC antibodies and DAPI, magnification ×10, scale bar = 100 µm (WT *n* = 7, TSP-1^−/−^ *n* = 7). In all results, WT are represented in black and TSP-1^−/−^ in white. All results are expressed in means ± sem. * *p* < 0.05, WT vs. TSP-1^−/−^.

To study the implication of the immune system in the early phase of flow-induced arterial remodeling, we focused on neutrophils (Figure 4a,c) and macrophages (Figure 4b,d), two myeloid cells recruited by TSP-1 which are involved in the flow-induced remodeling. In WT mice, macrophages and neutrophils accumulated around HF arteries, as evidenced by flow cytometry (Figure 4a,b) and immunostaining (Figure 4c,d,e). No significant macrophage and neutrophil accumulation occurred in HF TSP-1^−/−^ arteries (Figure 4c,e), confirming the role of tissue TSP-1 production previously observed in Figure 2c,d. 

**Figure 5:**
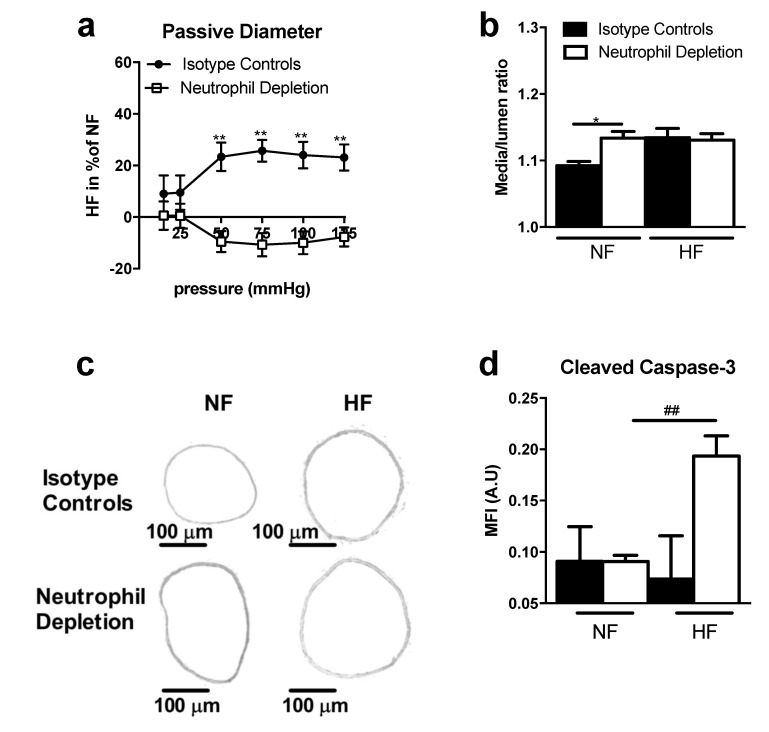
Remodeling study after seven days of chronic increase in blood flow and neutrophils depletion. Graphs show (**a**) the passive diameter of HF mesenteric arteries is percent of NF mesenteric arteries diameter and (**b**) the histomorphometry analysis of these mesenteric arteries as media-to-lumen ratio. (**c**) shows representative mesenteric arteries images (×10 magnification, scale bar = 100 µm). Neutrophil depleted mice (*n* = 8) are represented in white and isotype controls (*n* = 5) in black. (**d**) shows mesenteric arterial zone MFI measured of cleaved caspase-3 after four days of ligature in mesenteric arteries neutrophils depleted mice (*n* = 6) and isotype controls mice (*n* = 4). All results are expressed in means ± sem. * *p* < 0.05, ** *p* < 0.01 isotype controls vs. neutrophil depletion. ^##^ *p* < 0.01 NF vs. HF.

In order to identify the role of neutrophil in HF-remodeling, and because they initiate local pro-inflammatory processes, neutrophils were depleted in WT mice using anti-Ly6G antibody (Figure 5). Flow-mediated diameter enlargement failed to occur in neutropenic mice (Figure 5a,c), whereas significant remodeling was observed in mice treated with control isotype antibodies (Figure 5a,c). In control mice, the media-to-lumen ratio (Figure 5b and Supplementary Figure S3g,h) confirmed the increase in wall thickness in response to increased blood flow, whereas no change was observed in the neutropenic mice, suggesting a cross talk between TSP-1 and neutrophil for wall remodeling.

Four days after increasing blood flow, an immunofluorescence study was performed on arteries in order to determine the levels of vascular apoptosis, using cleaved caspase 3 staining as the marker (Figure 5d). Fluorescence intensity was significantly increased in HF arteries compared to NF arteries in neutrophil-depleted mice. Conversely, no statistical difference existed between HF and NF arteries in control mice. 

### 2.5. TSP-1 Deficiency Decreases Flow-Induced Inflammatory Gene Expression Levels

**Figure 6:**
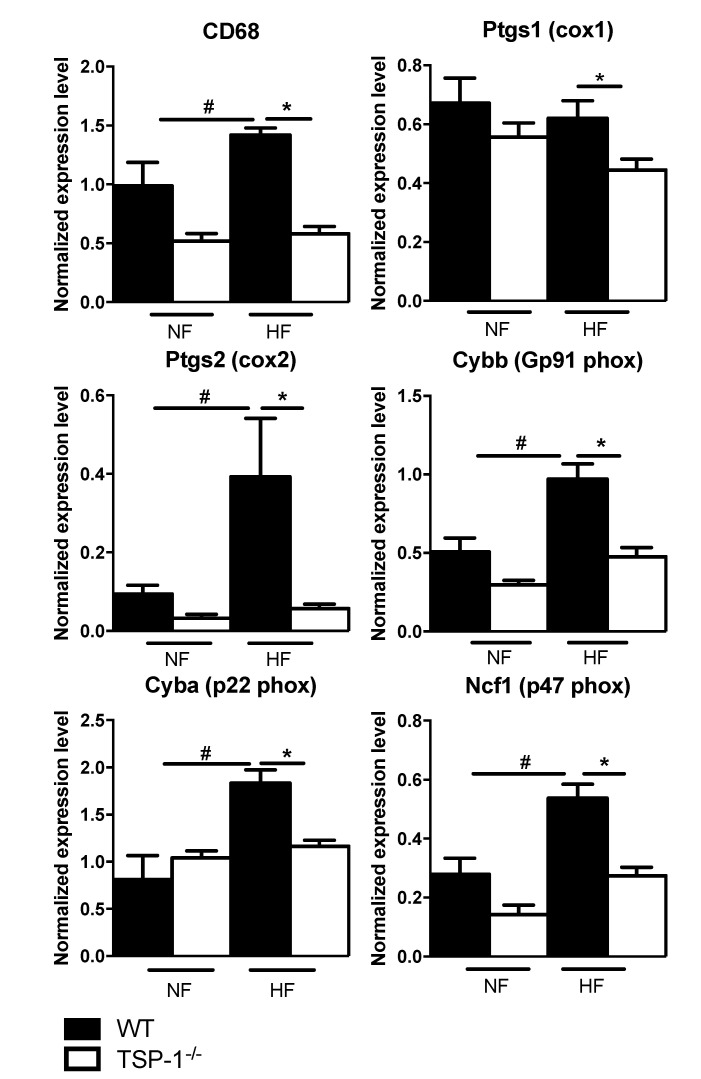
Gene expression study after qRT PCR experiments performed on WT and KO mesenteric arteries after 4 days of ligature. WT arteries (*n* = 6) are represented in black and TSP-1^−/−^ arteries (*n* = 5) in white. The genes studied are cd68, ptgs1, ptgs2, cybb, cyba, ncf1. Results are normalized to the hprt housekeeping gene and expressed as relative level of expression in means ± sem. * *p* < 0.05, WT vs. TSP-1^−/−^, ^#^ *p* < 0.05, NF vs. HF.

Studies have underlined the role of inflammation in the early phase of flow-mediated remodeling. We then determined inflammatory gene expression levels in NF and HF arteries, four days after surgery (Figure 6). As expected, the expression of the mRNA encoding *Cd68, P47^phox^, P22^phox^, Gp91^phox^* and *Ptgs2* were significantly increased in HF arteries from WT mice, compared to NF arteries. In contrast, no such increase occurred in TSP-1^−/−^ HF vessels, suggesting that TSP-1 may be involved in the flow-induced local inflammation.

## 3. Discussion

### 3.1. TSP-1 Is Essential for Small Vessel Remodeling during Adaptation to Blood Flow Increase

A chronic increase in arterial blood flow induces outward remodeling, thus allowing shear stress normalization [40]. Previous studies on cultured cells have linked rising blood flow to TSP-1 production [41]. Our aim was to investigate with in vivo experiments the role of TSP-1 in blood flow-induced remodeling of resistance arteries. 

We investigated in vivo flow-mediated remodeling using a model of chronic increase in arterial blood flow that does not affect systemic hemodynamics. The absence of flow-induced outward remodeling and compensatory wall thickening in TSP-1^−/−^ mice suggested that TSP-1 is strongly involved in this process. These observations and this conclusion remained valid one month after the increase in blood flow (Supplementary Figures S1a,b and S3i,j). We concluded that genetic TSP-1 ablation did not only delay remodeling but completely disabled it, pointing TSP-1 as a critical initiating factor of this process. Genetic TSP-1 depletion was associated with a decreased flow-induced dilation and with acetylcholine-induced relaxation by NO release. Indeed, the two other signaling pathways known to be triggered by acetylcholine (prostaglandin [42,43] and endothelium derived hyperpolarization factor [44]) were not affected by the absence of arterial TSP-1 (Supplementary Figure S2). This probably resulted from a decrease in the adaptation to genetic TSP-1 ablation; such a change in shear stress sensing and NO production will likely affect flow-mediated remodeling [9]. Hydralazine administration to TSP-1^−/−^ mice increased blood flow and shear stress in mesenteric arteries as expected [38,45,46], but failed to restore outward remodeling. We concluded that TSP-1 was not involved in flow sensing by the arteries, but in regulating the consequences of changes in blood flow. Indeed, independent pathways downstream of flow sensing appeared to be responsible for parietal remodeling. 

### 3.2. TSP-1 from Different Cellular Origins Impacts Small Vessel Remodeling in Different Ways

TSP-1 can be expressed by multiple types of circulating and tissue cells, including vascular smooth muscle and endothelial cells [14,15,16,17,18,19]. Our experiments suggested that TSP-1 secreted by circulating cells was essential for flow-mediated diameter expansion. Conversely, TSP-1 expressed by vascular cells was necessary for immune cells recruitment and wall thickening. 

### 3.3. TSP-1 Participates in Blood Flow-Mediated Small Vessel Remodeling by Recruiting Inflammatory Cells 

Outward remodeling is known to rely on the initial inflammatory response triggered by changes in blood flow, including limited but essential macrophage infiltration [31]. Here, genetic TSP-1 ablation drastically decreased vascular CD68 expression levels (a marker of myeloid immune cells, including monocytes/macrophages and neutrophils [47,48]) in HF arteries and, more particularly, reduced macrophage infiltration.

Genetic TSP-1 ablation also reduced the overall oxidative stress in the vessel wall (decreased *Gp91^phox^*, *p47^phox^* and *p22^phox^* expression) which is necessary for proper matrix metalloproteinase (MMP) activation, which is, in turn, needed for subsequent diameter expansion [49]. Moreover, the production of ROS by NADPH has previously been linked to neutrophil chemotaxis [50]. Neutrophil depletion completely prevented any significant outward remodeling or wall thickening of the HF artery. 

### 3.4. TSP-1 as a Therapeutic Target during Small Vessel Remodeling

Hypertension, obesity, the metabolic syndrome and exposure to glucose are all thought to enhance TSP1 expression, circulating or vascular [51,52]. Age will thus be associated to increased TSP1 expression, either directly or through co-morbidities. Conversely, TSP1 can enhance age-related deterioration of vascular function [53] and its dysregulation promotes angiogenesis in the eye [54]. The contribution of TSP1 to flow-mediated vascular remodeling strengthens the concept that novel therapeutic intervention to treat vascular remodeling should encompass an anti-inflammatory element. More specifically, novel pharmacological inhibitors of TSP1 and its receptors signaling (primarily CD47) [55,56] may help preserve vascular function in diseases associated to vascular remodeling and in age-related disorders.

## 4. Materials and Methods

### 4.1. Animal Models 

The protocol conformed with European Community standards on the care and use of laboratory animals (Authorization No. 00577) and with National Institutes of Health Guide for the Care and Use of Laboratory Animals (NIH Pub. No. 85-23, Revised 1996). The protocol was approved by the ethical committee (Permit No. CEEA PdL 2012.22).

For this study, mice lacking the gene thbs1, which encodes the TSP-1 protein, (tsp-1 knock-out; TSP-1^−/−^), were used [57,58], with wild-type (WT) litter mates as controls. Surgery was performed as previously described in order to modulate blood flow in mesenteric arteries [40,59]. Briefly, three consecutive first-order arteries, equivalent in apparent diameter, were used. Ligatures were applied to 4 s-order arterial branches. The artery located between the two ligated arteries was designed as HF artery [60]. Other mesenteric arteries located at distance from the ligated arteries were used as controls (normal flow, NF), so that each mouse had its own NF and HF arteries. For hydralazine experiments (a vasodilator compound), mice were given 200 mg/L hydralazine per day in tap water throughout the post-surgery period [38,46]. 

### 4.2. Pressure–Diameter Relationship in Mesenteric Arteries 

Arterial segments of mice were cannulated at both ends and mounted in a video monitored perfusion system (Living Systems, LSI, Burlington, VT, USA) as previously described [46]. Two glass cannulae were used to cannulate a 2–3 mm long arterial segment (SIV). Arterial segments were bathed in a 5 mL organ bath containing a Ca^2+^-free PSS containing ethylene-bis-(oxyethylenenitrolo) tetra-acetic acid (EGTA, 2 mmol/L) and sodium nitroprusside (SNP, 10 µmol/L). Pressure steps (10 to 125 mmHg) were then performed in order to determine passive arterial diameter. Furthermore, WT and TSP-1^−/−^ mice were treated during all the post-surgery period with hydralazine (200 mg/L in drinking water). Seven days after surgery, passive diameters were measured. Continuous pressure and diameter measurements were collected by our data acquisition system (BIOPAC MP100 and AcqKnowledge^®^ software; La Jolla, CA, USA) [36].

### 4.3. Histomorphometric Analyses

Mesenteric arterial segments pressurized at 75 mmHg and fixed in 4% para-formaldehyde solution were cut using a cryostat. Transverse sections (7 μm thick) were stained with orcein solution. After image acquisition (Olympus T100 microscope, Sony camera), internal and external medial circumference and surface were then measured and analyzed using the ImageJ software. Using these values, the CSA and wall thickness were calculated, as previously described [9]. The media-to-lumen ratio is calculated as the ratio of the external surface on the internal surface.

### 4.4. Quantitative Real-Time PCR

Guts were dissected out of mice four days after mesenteric ligature, placed in a 4 °C sterile PSS, and perivascular fat was carefully removed. Dissected arterial segments were kept in RNAlater^®^ buffer (Sigma-Aldrich, St Louis, MO, USA) at −20 °C. RNA extraction was performed in RNeasy^®^ Micro Kit (QIAGEN, Germantown, USA). Total RNA (100 ng) extracted from each artery were subjected to reverse transcription (RT) with the QuantiTect^®^ Reverse Transcription Kit (QIAGEN, Germantown, USA). Real-time PCR analyses of the target genes *Ccl2*, *Cd68*, *Itgam*, *Nfkbia*, *Rela*, *Ptgs1*, *Ptgs2*, *Ncf2*, *Ncf1*, *Cyba*, *Cybb*, *Mmp2*, *Mmp9*, *Timp1*, *Nos2*, *Nos3*, *Hif1a*, *Vegfa*, *Tgfb1*, *Tgfb2*, *Tgfb3*, *Ptpn6*, *Ppp1r12a*, *CD47*, *Akt1*, *Sod2* were performed. qPCR analysis was performed using a LightCycler 480 (Roche Life Science, Penzberg, Germany) and Power SYBR^®^ Green PCR Master Mix (Applied Biosystems, Illkirch, France). Gene-specific primers were designed using Primer3 online software and validated by PCR efficiency testing. A detailed list of primers and genes analyzed is provided in Supplementary Figure S5. Gene expression was quantified by the comparative cycle threshold method and *Hprt* gene expression was used as reference. 

### 4.5. Neutrophil Depletion

An intraperitoneal injection of an anti-murine Ly6G antibody (clone 1A8, BioXCell, Lebanon, USA) or control rat IgG2a isotype (clone 2A3, BioXCell) was performed 24 h before surgery. The injections were repeated every 72 h until sacrifice. The same amount (500 µg) of antibody and isotype was used for each injection [61]. 

### 4.6. Confocal Microscopy

Confocal imaging was performed on NF and HF arteries after ligation as previously reported [45]. Arterial segments were stained overnight (4 °C) with anti-mouse CD45-PE (clone 30-F11, rat IgG2bκ isotype, 1:200, BioLegend, San Diego, CA, USA), anti-mouse Ly-6G (Gr-1)-PE (clone RB6-8C5, rat IgG2bκ isotype, 1:200, eBioscience, San Diego, CA, USA), anti-mouse F4/80-PE (clone BM8, rat IgG2aκ isotype, 1:200, eBioscience) or with anti-cleaved caspase-3 Ab2 (1:100, Calbiochem, San Diego, CA, USA) and with F(ab’)2 donkey anti-rabbit IgG PE (1:100, 2 h, 20 °C, eBioscience). Samples were then washed with PBS three times for 5 min and mounted with Mowiol. Nuclei were stained with DAPI (Invitrogen, Waltham, MA, USA) 11 µg/mL. All dilutions were made in PBS-BSA 5%. Pictures were taken with a Nikon Eclipse TE2000S confocal microscope and analyzed with the MetaMorph^®^ software (San Jose, CA, USA).

### 4.7. Flow Cytometry Analysis 

Arteries without perivascular tissue were isolated and digested using 450 U/mL collagenase type I and 5 U/mL elastase for 1 h at 37 °C, with intermittent agitation. Cell suspensions were then passed through a 100 μm diameter filter before washing by centrifugation (800× *g*). Cells were fixed with 1% (*v*:*v*) PFA. After washing, cells were stained for 25 min at 4 °C with anti-Ly6G, anti-CD11b monoclonal antibodies (eBioscience) or control isotype antibodies. Cells were sorted by an LSR-II flow cytometer with the DIVA^®^ driving software (Becton Dickinson Biosciences, Franklin Lakes, NJ, USA). Data were analyzed using the FlowJo^®^ software (Tree Star, Inc., Ashland, OR, USA).

### 4.8. Mouse Irradiation and Adoptive Bone Marrow Transfer

Whole body irradiation of recipient mice (30 g) was conducted in a BIOBEAM 8000 gamma irradiator in order that mice received a total dose of 9.5 gray over 10 min. The mice were then allowed to rest for recovery. In each experiment, one irradiated mouse was kept free of transplantation as control. Its death within 2 weeks was considered a token of near complete bone marrow ablation. For adoptive bone marrow transfer, total bone marrow cells (BMC) were collected by flushing the dissected tibiae and humeri of euthanized donor mice with PBS until cells (red) were visually dissociated from bones (white). BMC were then dispersed with a pipette. Dense, solid tissue fragments were decanted for 30 s and eliminated in order to obtain a homogenous cell suspension. BMC collected from 2 donor mice were transplanted into 7 recipient mice by retro-orbital injection, 36 h after irradiation. Irradiated mice were then allowed to rest for a minimum of 2 weeks before experimentation. 

### 4.9. Statistical Analysis

Results were expressed as means ± SEM. Statistical analysis was performed using Prism software (v.8.0, GraphPad Software, San Diego, CA, USA). For vascular reactivity, a 2-way ANOVA for consecutive measurements was used followed by a Bonferroni post-test. A Mann–Whitney test was used for all other results. All results were considered as significantly different when *p* < 0.05.

## 5. Conclusions

**Figure 7:**
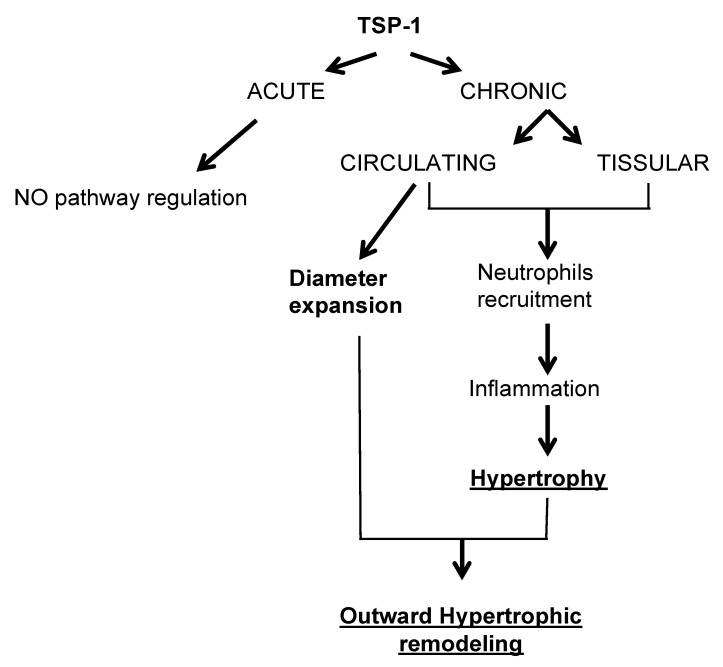
Schema of TSP-1 implication in mesenteric resistance arteries remodeling induced by a chronic increase in blood flow.

We demonstrated (Figure 7) that TSP-1 is a cornerstone of resistance artery remodeling in response to a chronic increase in blood flow. TSP-1 operates in two ways: Tissue-secreted TSP-1 triggers mainly neutrophil and macrophage recruitment, whereas circulating myeloid cell TSP-1 is responsible for the initiation of diameter expansion. This two-edged contribution during the acute phase of inflammation is necessary for any outward vascular remodeling to proceed. 

## Data Availability

The protocol conformed with European Community standards on the care and use of laboratory animals (Authorization No. 00577) and with National Institutes of Health Guide for the Care and Use of Laboratory Animals (NIH Pub. No. 85-23, Revised 1996). The protocol was approved by the ethical committee (Permit No. CEEA PdL 2012.22).

## Supplementary Materials

The followings are available online at https://www.mdpi.com/article/10.3390/ijms222112086/s1, Figure S1: Mesenteric arteries remodeling from WT and TSP-1^−/−^ mice after one month of chronic increase in blood flow, Figure S2 : Mesenteric arteries reactivity from TSP-1^−/−^ and WT mice, Figure S3 : Media thickness and cross section area of mesenteric arteries following flow induced remodeling and orcein staining, Figure S4 : Schematic representation of mesenteric arteries surgery and analysis, Figure S5 : list of primers and genes.

## Abbreviations

HF	High Flow
NF	Normal Flow
FMD	Flow-mediated dilation
CDR	Cumulative Dose Response
BM	Bone Marrow
MFI	Mean Fluorescence Intensity
TSP	Thrombospondin
WT	Wild Type
CD	Cluster of Differentiation
CSA	Cross Section Area
