# Supplementary material for: Dual Role of Thrombospondin-1 in Flow-Induced Remodeling
Source: Int J Mol Sci. 2021 Nov 8;22(21):12086. doi: 10.3390/ijms222112086 (PMC8584526; doi:10.3390/ijms222112086)
Supplement: Supplementary file 1 [file ijms-22-12086-s001.zip › ijms-1341703-supplementary.pdf]

Supplementary data

S1

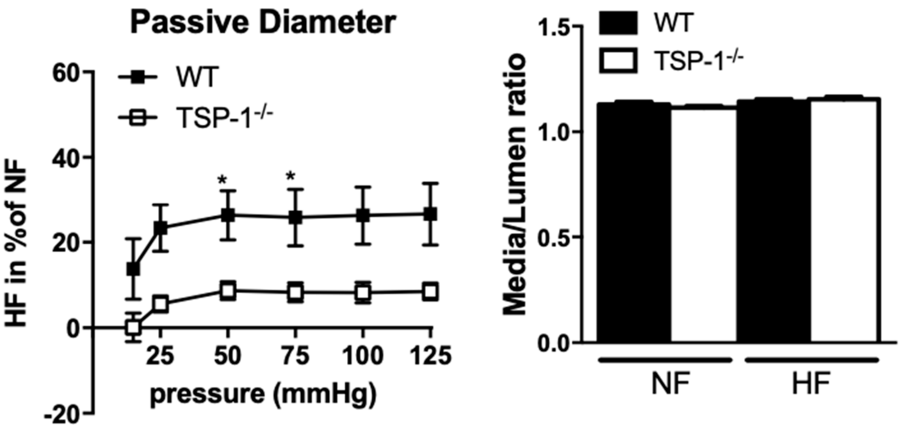

Figure S1 Mesenteric arteries remodeling from WT and TSP-1$^{-/-}$ mice after one month of chronic increase in blood flow

Graphs show (a) the passive diameter of HF arteries in percentage of NF arteries, (b) the histomorphometry analysis of these arteries as Wall-to-lumen ratio. The TSP-1$^{-/-}$ (n=6) arteries are represented in white, the WT (n=7) in black. All results are expressed in means ± sem. * $p<0.05$ WT vs TSP-1$^{-/-}$.

S2

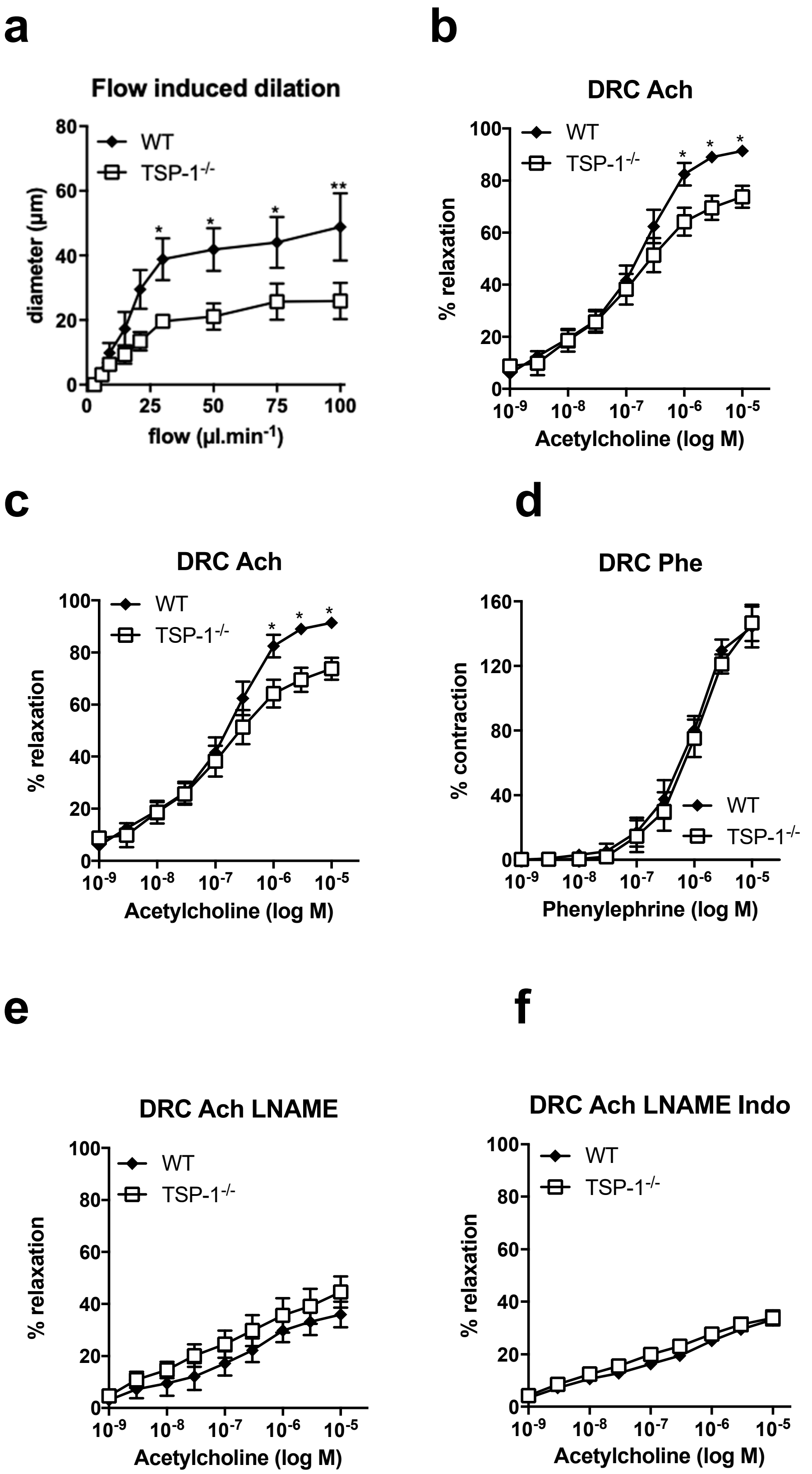

Figure S2 Mesenteric arteries reactivity from TSP-1$^{-/-}$ and WT mice

S3

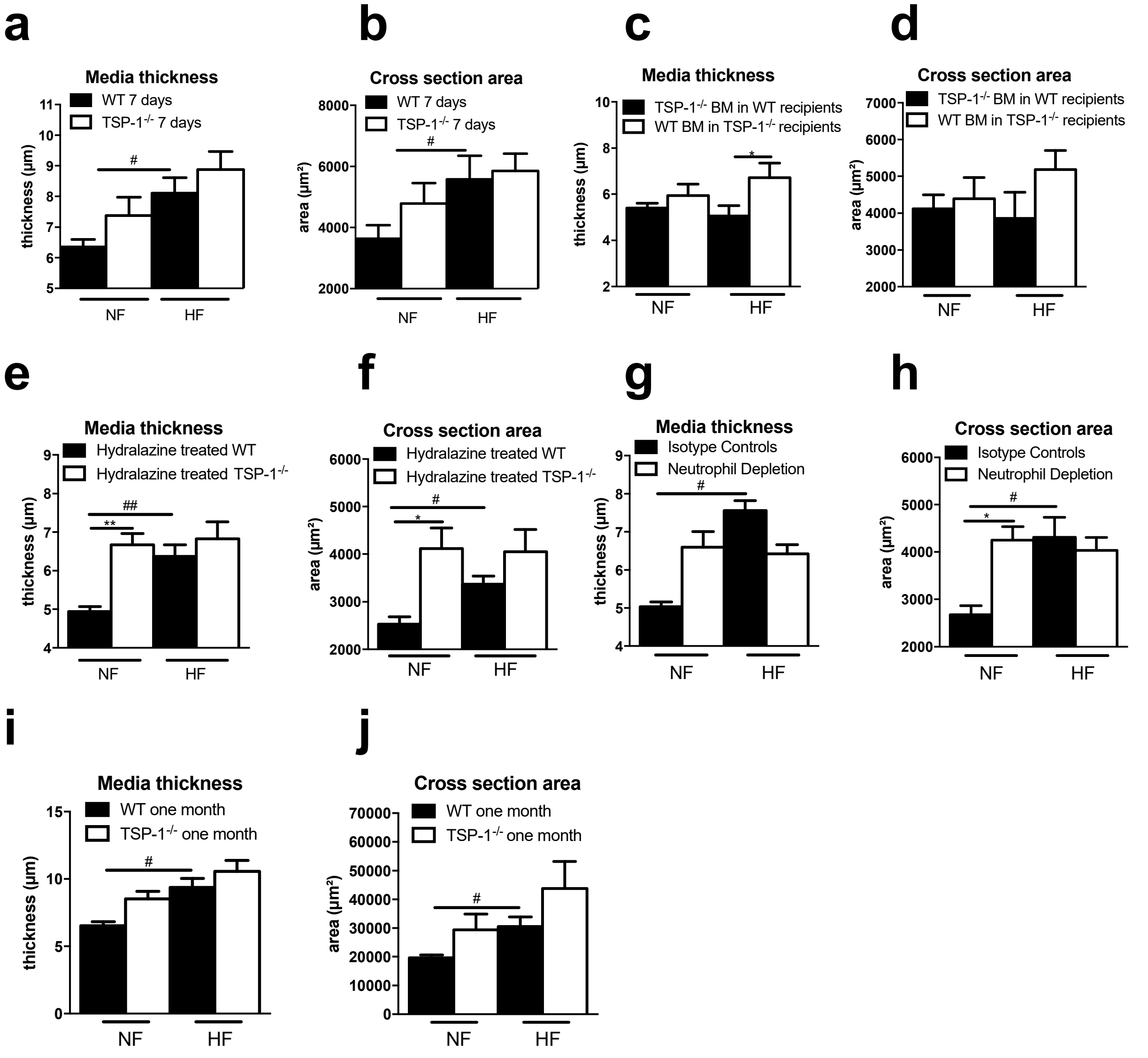

Figure S3 Media thickness and cross section area of mesenteric arteries following flow induced remodeling and orcein staining

Graph (a) and (b) show respectively the media thickness and the cross section area of WT (n=5) and TSP-1$^{-/-}$ (n=4) normal flow(NF) or high flow(HF) arteries after 7 days of chronic increase in blood flow. (c) and (d) represent the media thickness and the cross section area of NF and HF arteries from TSP-1$^{-/-}$ BMC in WT recipients (n=5) and WT BMC in TSP-1$^{-/-}$ recipients (n=8) after 7 days of remodeling. (e) and (f) represent the media thickness and the cross section area of WT (n=5) and TSP-1$^{-/-}$ (n=8) NF and HFarteries after 7 days of treatment with hydralazine during the remodeling experiment. (g) and (h) represent the media thickness and the cross section area of isotype controls (n=4) or neutrophil depletion (n=7) NF or HF arteries after 7 days of remodeling.

(i) and (j) represent the media thickness and the cross section area of WT (n=5) and TSP-1$^{-/-}$ (n=5) arteries after one month of remodeling induced by a chronic increase of blood flow. # $p<0.05$ NF vs HF, ## $p<0.01$ NF vs HF, * $p<0.05$ TSP-1$^{-/-}$ BMC in WT recipients vs WT BMC in TSP-1$^{-/-}$ recipients, hydralazine treated WT vs hydralazine treated TSP-1$^{-/-}$, isotype controls vs neutrophil depletion, **$p<0.01$ hydralazine treated WT vs hydralazine treated TSP-1$^{-/-}$. Results are expressed in means ± sem.

S4

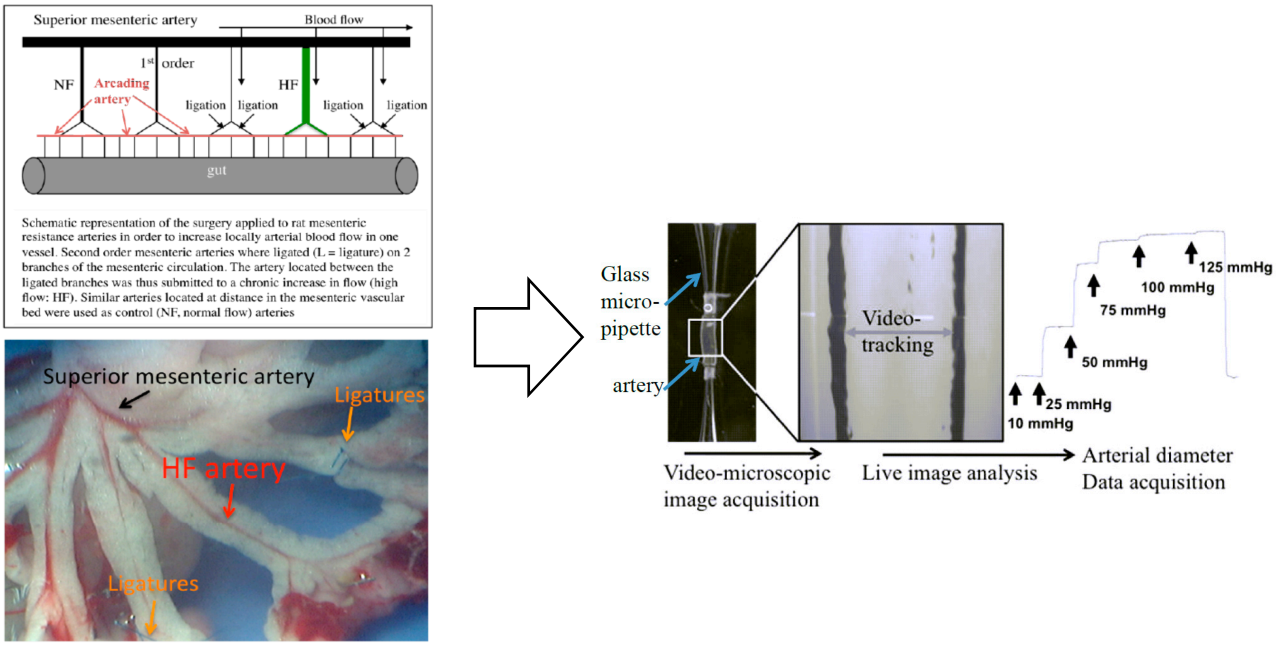

Figure S4 Schematic representation of mesenteric arteries surgery and analysis

S5

Gene	NCBI Reference Sequence	Protein	forward sequence (5'-3')	reverse sequence (5'-3')
Ccl2	NM_011333.3	MCP-1	gcctgctgttcacagttgc	caggtgagtggggcgtta
CD68	NM_009853.1	CD68	gacctacatcagagcccgagt	cgccatgaatgtccactg
Itgam	NM_001082960.1	CD11b	caatagccagcctcagtgc	gagcccaggggagaagtg
Nfkbia	NM_010907.1	Ikkb	acgagcaaatggtgaaggag	atgattgccaagtgcagga
Rela	NM_009045.4	p65-Nfkb	cccagaccgcagtatccat	gctccaggtctcgcttctt
Ptgs1	NM_008969.3	cox1	cctctttccaggagctcaca	tcgatgtcaccgtacagctc
Ptgs2	NM_011198.3	cox2	gatgctcttccgagctgtg	ggattggaacagcaaggattt
Ncf2	NM_010877.4	p67phox	ccaagacaacttttctggcttc	ttctggggttttgggtctg
Ncf1	NM_010876.3	p47phox	gaggttgggtccctgcat	gctttgatggttacatacggttc
Cyba	NM_007806.3	p22phox	tgccctccacttcctgtt	gcagatagatcacactggcaat
Cybb	NM_007807.4	Gp91phox	gaggttggttcggttttgg	gttttgaaagggtgggtgac
Mmp2	NM_008610.2	Mmp2	gtgggacaagaaccagatcac	gcatcatccacggtttcag
Mmp9	NM_013599.2	Mmp9	ttctggcacacgcctttc	ccatagtaagtggggatcacg
Timp1	NM_011593.2	Timp1	catggaaagcctctgtggat	gatgtgcaaatttccgttcc
Nos2	NM_010927.3	iNOS	ctttgccacggacgagac	tcattgtactctgagggctgac
Nos3	NM_008713.4	eNOS	ccagtgccctgcttcatc	gcagggcaagttaggatcag
Hif1a	NM_010431.2	Hif1a	gcactagacaaagttcacctgaga	cgctatccacatcaaagcaa
Vegfa	NM_001025250.3	Vegfa	aaacgaaagcgcaagaaatc	atgctttctccgctctgaac
Tgfb1	NM_011577.1	Tgfb1	tggagcaacatgtggaactc	cagcagccggttaccaag
Tgfb2	NM_009367.3	Tgfb2	tcttccgcttgcaaaacc	gtgggagatgttaagtctttgga
Tgfb3	NM_009368.3	Tgfb3	ccctggacaccaattactgc	tcaatataaaggggcgtaca
Ptpn6	NM_013545.2	SHP1	ggcccatcattgtgcatt	ctggatatcaatgtcacagtctagc
Ppp1r12a	NM_027892.2	Mypt	ggcgacccagagaaaagc	tgccgctcttgctcattt
Cd47	NM_010581.3	CD47	ttgttggagccatccttctc	caatgaggccaagtccaga
Akt1	NM_009652.2	Akt1	tcgtgtggcaggatgtgtat	acctggtgtcagtctcagagg
Sod2	NM_013671.3	MnSOD	gacccattgcaaggaacaa	gtagtaagcgtgctcccacac

Figure S5 : list of primers and genes
